# Development of a Novel Catalytic Membrane Reactor for Heterogeneous Catalysis in Supercritical CO_2_

**DOI:** 10.3390/ijms11010164

**Published:** 2010-01-13

**Authors:** Nazrul M. Islam, Maya Chatterjee, Yutaka Ikushima, Toshiro Yokoyama, Hajime Kawanami

**Affiliations:** 1 Research Center for Co MPact Chemical Process, National Institute of Advanced Industrial Science and Technology (AIST), 4-2-1 Nigatake, Miyagino-Ku, Sendai, 983-8551, Japan; E-Mails: islam.n.ab@m.titech.ac.jp (N.M.I.); c-maya@aist.go.jp (M.C.); y-ikushima@aist.go.jp (Y.I.); 2 Department of Organic and Polymeric Materials, Tokyo Institute of Technology, 2-12-1-S5-20, Ookayama, Meguro-ku, Tokyo 152-8552, Japan

**Keywords:** catalytic membrane reactor, supercritical carbon dioxide, mesoporous silica membrane, selective hydrogenation, cinnamaldehyde

## Abstract

A novel type of high-pressure membrane reactor has been developed for hydrogenation in supercritical carbon dioxide (scCO_2_). The main objectives of the design of the reactor are the separate feeding of hydrogen and substrate in scCO_2_ for safe reactions in a continuous flow process, and to reduce the reaction time. By using this new reactor, hydrogenation of cinnamaldehyde into hydrocinnamaldehyde has been successfully carried out with 100% selectivity at 50 °C in 10 MPa (H_2_: 1 MPa, CO_2_: 9 MPa) with a flow rate of substrate ranging from 0.05 to 1.0 mL/min.

## 1. Introduction

Supercritical carbon dioxide (scCO_2_) is an environmentally benign reaction medium because of its unique properties compared to conventional organic solvents, such as high diffusivity, low viscosity and high miscibility with gases, such as H_2_, O_2_ *etc*. These physical properties can be easily tuned by adjusting the pressure and temperature, and various reactions have been accelerated with high yields and selectivities [1]. Despite the many advantages of homogenous catalytic reactions in scCO_2_, the difficulties of the product-catalyst separations still remain.

To overcome these difficulties, a flow reaction system in the presence of heterogeneous catalyst in scCO_2_ has been developed [2], and an inorganic membrane scCO_2_ reactor has been reported [3–6]. Membrane reactors are multifunctional reactors with compact design and are currently being applied to various chemical reactions in both the academic and industrial fields [7]. The membranes used have structures with small pores and are thus known as nanofiltration membranes. They allow the easy separation of organic molecules and catalyst by passing through the small pores. Generally, organic membranes are used for various organic syntheses. However, these membranes face several shortcomings, like swelling, lack of stability and unsuitability for high pressure conditions. On the other hand, various types of inorganic membrane have been developed, some of which are also available for high pressure conditions [8–10]. Inorganic membranes exhibit physical and chemical properties are comparable or better than those of organic membranes, including a better structural stability and no problems of swelling or compaction. Moreover, a silica membrane showed high H_2_ permeance over CO_2_ [11]. This approach has been successfully applied to a system in scCO_2_ medium in the presence of homogeneous catalysis. However, those methods still have some disadvantages like low solubility of homogeneous catalysts in scCO_2_ and to increase the solubility, expensive ligands such as perfluoroalkyl (low cohesive energy density) are necessary [12].

Here, we have developed a new membrane reactor containing a mesoporous silica membrane (inorganic membrane) supported on α-alumina with a heterogeneous catalyst in scCO_2_ and demonstrated the hydrogenation of cinnamaldehyde (CAL) into hydrocinnamaldehyde (HCAL) or hydrocinnamylalcohol (HCOL). The objective of the present method was to develop a membrane reactor that can be used for reactions in scCO_2_ to achieve good selectivity of the desired products.

## 2. Experimental Section

### 2.1. Materials

All the chemicals used in this study were of reagent grade. P123 copolymer was received as a sample complements of BASF, cetyltrimethylammonium bromide (CTABr), PdCl_2_ and *trans*-cinnamaldehyde (99%) were obtained from Aldrich. Tetraethylorthosilicate (TEOS, 95%), HCl, and NaOH were from Wako Pure Chemicals Co. Ltd. Hydrogen (99.99%) and carbon dioxide (>99.99%) were supplied by Nippon Sanso. Co. Ltd.

### 2.2. Construction of a Catalytic Membrane Flow Reactor

#### 2.2.1. Schematic diagram of the reactor

**Figure 1.**
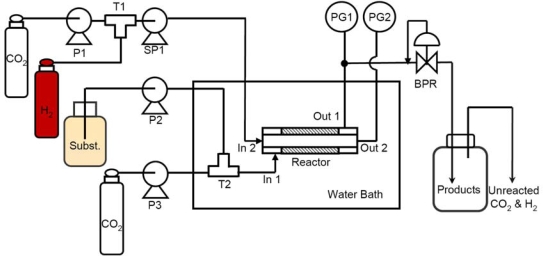
Schematic diagram of the catalytic membrane reactor. SP1: Syringe pump; P1: CO_2_ delivery pump; P2: Reciprocating liquid pump; P3: CO_2_ delivery pump; T1 & T2: T-shape mixer, Reactor: High-pressure membrane reactor, In 1: Inlet of outer tube of Reactor; In 2: Inlet of inner tube of Reactor, Out 1: Outlet of out tube of Reactor; Out 2: Outlet of inner tube of Reactor; R2: Reaction Chamber, R3: Outlet compartment of the reaction chamber; PG1: Pressure Gauge for outer tube of Reactor, PG2: Pressure Gauge for inner tube of Reactor; BPR: Back Pressure Regulator.

**Figure 2.**
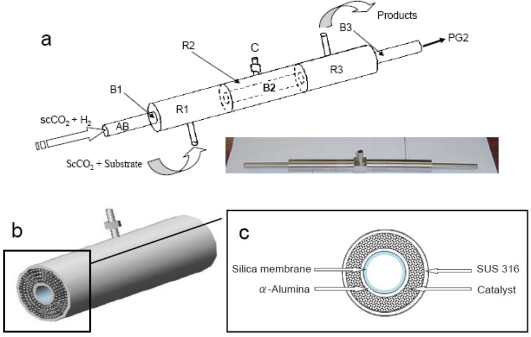
Modeled diagram of the catalytic membrane flow reactor; (a) Whole reaction unit (with photo) and (b) Reaction zone, where reaction takes place. (c) Cross section of reaction zone.

Figure 1 shows the schematic flow model of the novel catalytic membrane flow reactor (CMFR). The cylindrical reaction unit of the CMFR is placed in a constant temperature water bath to maintain the reaction temperature. A detailed description of the reactor unit is given described in the following section. Hydrogen gas is introduced into the syringe pump SP1 (ISCO DX260), then CO_2_ added into the syringe pump SP1 up to the desired pressure by JUSCO 880 PU CO_2_ delivery pump P1. Syngas (H_2_ + CO_2_) was delivered into the inner tube of the membrane reactor (AB in Figure 2a) from In 1. The substrate stream from high pressure pump P2 was mixed at T2 with scCO_2_ delivered by P3 (JUSCO CO_2_ delivery pump), then introduced into the membrane reactor through In 1. Pressure gauges PG1 and PG2 are used to monitor the exact pressure of the reaction unit. Total pressure was controlled by a JUSCO Back Pressure Regulator (BPR). Finally, the product was collected in the trap which is cooled at 0 °C by ice. Product yields were obtained by GC analysis (Varian CP-3800) with tridecane as a standard, and the identities of all products were confirmed by GC-MS (Varian CP-3800-1200L), using authentic samples for comparison.

When the reaction was started, the inside and outside pressures of membrane reactor should be maintained almost equally, then the pressures should be increased gradually and carefully. Because membrane reactor is easily broken, and silica membrane can be peeled off by large differences of pressure, which might cause leakage of H_2_ and CO_2_ from the membrane reactor part. According to the safety regulations, pure hydrogen gas never introduced into the inner tube directly, and hydrogen gas should be diluted with CO_2_.

#### 2.2.2. Reaction unit

For better clarification of the reaction unit, a schematic diagram of membrane reactor is presented in Figure 2. As shown in Figure 2a, the reactor consists of two cylindrical tubes, the inner tube (AB; i.d. 1 cm) being covered with an outer tube (i.d. 3 cm). The outer tube is made of SUS 316 stainless steel; divided in to three chambers R1, R2, and R3. It has to be mentioned that, the inner tube comprises of three sections B1, B2 and B3, each of those are 10 cm in length. Both B1 and B3 are made of same material (SUS 316) differing from that of B2, which is made of macroporous α-Al_2_O_3_, where a macroporous α-Al_2_O_3_ membrane is usually used as support layer having no separation capability [13]. The tube B2 connects B1 and B3 on its both sides comprising a total tube length of 30 cm (inner tube). An enlarged version of the main part of CMFR made up by B2 and R2 is shown in Figure 2b.

**Figure 3.**
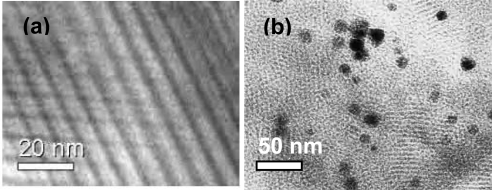
(a) TEM image of the mesoporous silica installed on the inner wall of the inner tube (B2) of membrane reactor; (b) TEM image of Pd/MCM-41 catalyst having pore size 3.52 nm, surface area 981 m^2^/g, and Pd ~1 wt %.

**Figure 4.**
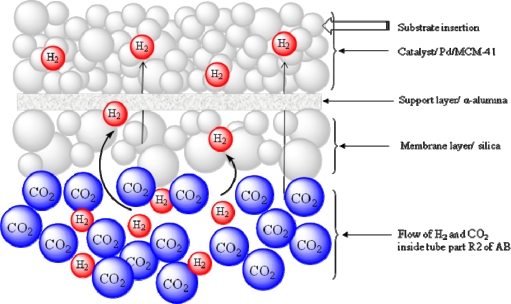
Diagram of hydrogen diffusion process from inner tube to catalytic bed through the membrane.

A mesoporous silica membrane was fabricated on the inner wall of the α-Al_2_O_3_ support (tube B2). In the case of the outer tube, two stainless steel nets are connected between R1-R2 and R2-R3. The space between the inner tube (B2) and outer tube (R2) was filled with the catalyst through the port C, and closed securely to prevent any leakage of reactant gases. The pore sizes of the nets are maintained in such way that the catalysts can be retained in the reaction chamber letting the product go out from the system through the outlet attached with chamber R3. Figure 2c shows the cross section of the main part of CMFR (Figure 2b). Figure 3a shows the TEM image of the mesoporous silica membrane that was installed inner wall of the inner tube, and Figure 3b shows the TEM image of Pd/MCM-41 catalyst whose pore size is 3.52 nm, surface area is 981 m^2^/g, and loaded Pd (nano particle) is ~1 wt %. Figure 4 shows the gas diffusion processes through the membrane. The premixed H_2_ and CO_2_ gases are being pumped by a high pressure pump P1 to the inner tube B2. The silica membrane is selective to the H_2_ gas and allows diffusion through porous silica membrane toward the catalyst chamber, which is one of the most significant points of this new reactor system. After entering into the catalytic bed H_2_ activates the metal catalyst active sites of the catalyst and reacts with substrate. The product is then collected through a back pressure regulator (BPR) using a cooled trap. In this newly developed reactor, any H_2_ selective porous inorganic membrane, such as zeolite membrane, alumina-silicate membrane, silica membrane or composite membrane can be used successfully.

#### 2.1.3. Preparation of the mesoporous silica membrane on α-Al_2_O_3_ support

The mesoporous silica membrane has been prepared by a typical hydrothermal method [14]. Briefly, 10 g of triblock copolymer P123, is dissolved in 175 g water containing 15 mL of HCl (1M) and the resulting solution is stirred at 35 °C for 1 h. After that TEOS is added to the above solution and the resulting silica sol solution is stirred again vigorously for 1 h. The mole ratio of the resultant gel is 175 H_2_O: 1:0 P123: 0.017HCL: 1 TEOS. The silica gel is then introduced inside the α-Al_2_O_3_ support. Finally, the membrane is dried for 48 h at room temperature. To remove the structure directing surfactant, the as-made mesoporous silica membrane is calcined at 450 °C for 6 h. The resultant membrane is characterized by powder X-ray diffraction pattern (XRD; not shown) and transmission electron microgram (TEM). Figure 3a shows the TEM image of the membrane taken by scrapping some sample from the tube, which exhibits a regular pattern of hexagonal mesoporous structure.

#### 2.1.4. Catalyst synthesis

Pd catalyst supported on MCM-41 (~1 wt % Pd) has been prepared by a typical hydrothermal method [15]. Pd salt solution is added to CTABr solution containing NaOH. TEOS is added dropwise to the above solution with stirring for 1 h. The resulting gel is then loaded into Teflon-lined stainless-steel autoclave and heated at 100 °C for 72 h in static condition. The solid product is recovered by filtration, washed with water and dried at ambient temperature. The occluded organic material is removed by calcined at 550 °C for 10 h.

## 3. Results and Discussion

### 3.1. Test of Catalytic Hydrogenation in the CMFR

**Scheme 1.**
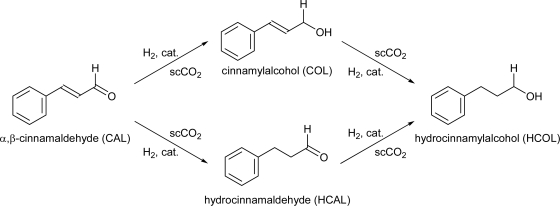
Hydrogeneration of cinnamaldehyde (α,β-unsaturated aldehyde) in scCO_2_.

In order to test the performance of the newly developed membrane reactor, we have attempted the selective hydrogenation of cinnamaldehyde (an α,β unsaturated aldehyde) in scCO_2_ as a model reaction (Scheme 1). The reaction was carried out using Pd-MCM-41 (Pd ~1 wt %) catalyst at 50 °C in the pressure range of 9–16 MPa of CO_2_ and 1–1.5 MPa H_2_ with substrate flow rate of ranging from 0.05 to1.0 mL/min.

### 3.2. Phase Diagram

**Figure 5.**
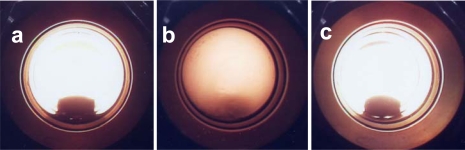
Phase behavior studies with CAL at 50 °C, 1 MPa H_2_ and (a) 6 MPa (Total 7 MPa), (b) 9 MPa (Total 10 MPa), and (c) 12 MPa (Total 13 MPa) of CO_2_ pressure.

Before the reaction, the phase behavior was studied with a high-pressure view cell (cell volume: 10 mL attached sapphire windows), as it is an important concern for a better understanding of the operation of the catalytic system in scCO_2_. The solubility of CAL in a mixture of CO_2_ and H_2_ is estimated from the phase behavior inspection at 50 °C. From the visual observation, it has been found that at constant H_2_ pressure of 1 MPa, the solubility of CAL increases with increasing CO_2_ pressure from 6 MPa to 12 MPa and a homogeneous phase is obtained at above 12 MPa, and transition phase from gas-liquid two-phase to a supercritical phase (single) at 9 MPa of CO_2_ was observed (Figure 5).

### 3.3. Test Reaction

The probable reaction pathway under the studied reaction conditions is shown in Scheme 1 [16,17]. Table 1 shows the results along with the experimental conditions. According to the primary results, hydrocinnamaldehyde (HCAL) is the major product and the highest selectivity of 100% (entry 1) is obtained at 9 MPa of CO_2_, 1 MPa partial pressure of H_2_ and the flow rate of substrate is 0.1 mL/min.

At the higher pressure of CO_2_ above 12 MPa of supercritical phase, the fully hydrogenated compound, HCOL, was also observed, and HCOL selectivity was showed the maximum at 14 MPa (total pressure: 15 MPa). Usually, Pd catalysts exhibit high selectivity for the hydrogenation of the C=C double bond in scCO_2_ [16,17]. The present study shows that the higher selectivity of HCAL by the hydrogenation of C=C double bond can be attributed to the diffusion of H_2_ through the membrane and its reaction with the substrate successfully at around 9 MPa of transition phase. Furthermore, as the flow rate and retention time decrease (Entry 3) the conversion increases, but formation of the saturated compound 3-phenylpropanol (HCOL) was observed. This result indicates that as H_2_ is readily available on the catalyst surface, HCAL, which was formed previously, is converting to HCOL. Moreover, no reaction has taken place in the absence of membrane on the inner wall of the α-alumina support under the same reaction conditions as mentioned in the Table 1. So, it could be concluded that the membrane is functionally active and playing an important role to carry out the hydrogenation reaction of CAL. Our future plans are to extend the use of this type of reactor to other different types of reaction by varying the membrane. Thus, the combination of a suitable membrane along with scCO_2_ might be one of the key tools to enhance the selectivity of the reaction.

## 4. Conclusions

We developed a high-pressure catalytic membrane reactor for continuous flow reactions in scCO_2_. The reactor was preliminarily tested with the hydrogenation of cinnamaldehyde. A 100% selectivity of HCAL from cinnamaldehyde was achieved at 10 MPa of the total pressure (H_2_: 1 MPa, CO_2_: 9 MPa, 50 °C) after 30 min of reaction time. The use of this type of membrane reactor provides several advantages such as short reaction time, easy product separation from reactant, control and prevention of side reactions, *etc*. This high-pressure membrane reactor could offer attractive research opportunities, not only to the field of membrane reactors but also in heterogeneous catalysis in scCO_2_. For future work, we will improve the reactor to scale-up the productivity from laboratory scale to the industrial scale for industrial approaches.

## Figures and Tables

**Table 1.** Hydrogenation of cinnamaldehyde in scCO_2_ at 50 °C

**Entry**	**P_H2_ (MPa)**	**P_CO2_ (MPa)**	**Flow CAL mL/min**	**Time (min)**	**Conversion (%)**	**Selectivity (%)**		
						**HCAL (%)**	**COL (%)**	**HCOL (%)**
1	1	9	0.1	30	1.2	100	0	0
2	1	9	0.1	60	0.3	79	0	21
3	1	9	0.05	15	2.7	74	0	26
4	1	12	0.05	60	3.3	69	0	31
5	1	12	0.05	60	4.5	59	0	41
6	1.5	14	0.05	60	5.3	58	0	40
7	1.5	16	0.05	60	5.8	65	0	35
